# Directionality in protein fold prediction

**DOI:** 10.1186/1471-2105-11-172

**Published:** 2010-04-07

**Authors:** Jonathan J Ellis, Fabien PE Huard, Charlotte M Deane, Sheenal Srivastava, Graham R Wood

**Affiliations:** 1 Department of Statistics, Macquarie University, Sydney, NSW 2109, Australia; 2 Department of Statistics, Oxford University, 1 South Parks Road, Oxford OX1 3TG, UK

## Abstract

**Background:**

Ever since the ground-breaking work of Anfinsen et al. in which a denatured protein was found to refold to its native state, it has been frequently stated by the protein fold prediction community that all the information required for protein folding lies in the amino acid sequence. Recent in vitro experiments and in silico computational studies, however, have shown that cotranslation may affect the folding pathway of some proteins, especially those of ancient folds. In this paper aspects of cotranslational folding have been incorporated into a protein structure prediction algorithm by adapting the Rosetta program to fold proteins as the nascent chain elongates. This makes it possible to conduct a pairwise comparison of folding accuracy, by comparing folds created sequentially from each end of the protein.

**Results:**

A single main result emerged: in 94% of proteins analyzed, following the sense of translation, from N-terminus to C-terminus, produced better predictions than following the reverse sense of translation, from the C-terminus to N-terminus. Two secondary results emerged. First, this superiority of N-terminus to C-terminus folding was more marked for proteins showing stronger evidence of cotranslation and second, an algorithm following the sense of translation produced predictions comparable to, and occasionally better than, Rosetta.

**Conclusions:**

There is a directionality effect in protein fold prediction. At present, prediction methods appear to be too noisy to take advantage of this effect; as techniques refine, it may be possible to draw benefit from a sequential approach to protein fold prediction.

## Background

The purpose of this paper is to investigate whether directionality of synthesis can have an impact on the accuracy of protein structure prediction. In order to do this a sequential structure prediction algorithm, based on the most successful free modelling method of our time, Rosetta, was developed and used to predict structure, first starting from the nitrogen terminus and then starting from the carbon terminus. Free modelling protein structure prediction methodology has improved in recent years, but is still not accurate enough to be considered satisfactory (see results of CASP6 [1] and CASP7 [2,3] and the more recent CASP8 [4]). Given this noisy nature of current free modelling stucture prediction techniques, the pairwise comparison design used here appears to be required; it succeeded in detecting a consistent directionality effect. We begin, however, by summarizing the area.

Almost fifty years ago Anfinsen et al. [5,6] showed that denatured small globular proteins could refold to their native state. On the other hand, experimentalists have known for many years that cotranslation can play an important role in protein folding [7-12]. Polypeptides are synthesized sequentially, and translation can occur at variable rates according to codon speed [13-17]. In *Escherichia coli*, for example, translation can occur in the order of 0.05 s/codon [13,18-20]. On the other hand, it has been shown that helices and sheets fold in the low millisecond scale [21-23]. Therefore, some proteins fold faster than they elongate, and it is reasonable to assume that nascent chains can adopt secondary or tertiary structures cotranslationally. Experimental evidence for cotranslational folding dates back to the 1960s with a study on cotranslation in vivo reporting that ribosome-bound *β*-galactosidase was showing enzymic activity [24]. More recently it has been shown that the Semliki Forest Virus Protein (SFVP), which contains a protease domain that folds to autocatalytically cleave the protein from a larger polyprotein precursor, gains its enzymic activity before complete synthesis of the polyprotein [25]. Moreover, the rapid cotranslational folding of SFVP does not require additional cellular components [26].

In addition to enzymatic activity whilst still bound to the ribosome, intermediate stages of cotranslational folding may have native-like structures. Various length *α*-globins have been shown to have specific heme binding activity on several truncated ribosome-bound nascent chains. The shortest of these contained only the first 86 residues (from a total of 147 residues), demonstrating that the nascent chain has native-like structure [27]. NMR studies of nascent chains containing tandem Ig domains and still attached to the ribosome revealed that the N-terminus domain folds to its native state while the C-terminus domain is largely unfolded and flexible [28]. Recent molecular dynamics simulations also conclude that small peptides may adopt a conformation that is similar to the one adopted in full proteins [29]. The discovery of the formation of disulphide bonds in nascent immunoglobulin peptides also confirms the ability of proteins to begin to fold whilst they are being synthesized [30,31].

As well as adopting native-like conformations while still attached to the ribosome, there is evidence that peptides can begin to fold whilst still in the ribosomal exit tunnel. Analysis of the ribosomal exit tunnel reveals that peptides can traverse the tunnel in an *α*-helical conformation [32], but that at no point is the tunnel big enough to accommodate structures larger than *α*-helices [33,34]. Peptides are not restricted to an *α*-helix, however, and may adopt more extended conformations [35]. Analysis of the exit tunnel has also shown that the tunnel can entropically stabilize *α*-helical conformations as they pass through [36].

The rate of in vitro refolding has often been observed to be slower than the corresponding rate in vivo [37,38]. Cotranslation has been studied in the bacterial luciferase *αβ *heterodimer, and the formation of the heterodimer is faster when the *β *monomer is translated in the presence of the folded *α *monomer than when the *β *monomer is refolded from a denatured state [38]. This shows that, under cotranslational folding, the *β *monomer is able to obtain a conformation that is more receptive to the formation of the dimer, thus avoiding kinetic traps associated with refolding from a denatured state [39]. Native-like structure has also been observed in cotranslationally folding monomeric firefly luciferase; again, cotranslational and in vitro folding pathways appear to be different, with cotranslational folding being faster [40]. Cotranslational folding in P22 tailspike protein has been shown to guide the peptide away from aggregation-prone conformations that are frequently encountered when refolding in vitro, leading to the hypothesis that cotranslational folding could be an efficient strategy for the folding of *β*-sheet topologies, and for large, multidomain proteins in general [41]. One possible explanation for this is that the peptide begins to fold while still attached to the ribosome [42,43]. Another possible explanation is the existence of additional folding machinery contained in the cell; however, only approximately 20% of proteins associate, for example, with chaperones [44,45]. The removal of major chaperones, such as DnaK and Hsp70, in *E. coli *has no adverse effect on cell growth or viability [46,47]. This suggests that chaperones alone cannot account for the higher folding rates observed in vivo.

Complementing these experimental findings, computational models of cotranslational folding have also been explored, an early, incidental, use of this idea appearing in [48]. Simple computational models of protein folding incorporating cotranslation demonstrate that such folding favours local contacts in intermediate and final folds [49,50]. More recently the effect of energy barriers on simple cotranslational models was studied, and it was found that the ground state of proteins folded sequentially was not necessarily the one of lowest energy [51]. Computational models have provided evidence that nascent chains may adopt partial structures similar to the corresponding parts of the complete protein [52]. Other lattice studies present a differing view of cotranslation where nascent peptides can remain largely unstructured until the final stages of synthesis (estimated to be when 90% or more of the protein has been extruded) [53]. This finding is dependent on the involvement of the C-terminal in tertiary interactions, and may not be applicable to all proteins. There is also evidence arising from lattice models that cotranslational folding pathways and refolding pathways are different [53]. Computational simulations of real proteins folding cotranslationally compared to refolding from a denatured state show mixed results. Chymotrypsin inhibitor 2 (CI2) and barnase were shown to fold mostly posttranslationally, with intermediates similar to those observed in refolding [54]. An alternative computational, cotranslational approach using dynamic optimisation in [55] found that major elements of the CI2 tertiary structure only form when the amino acid string is fully translated. For SFVP, which is known to fold cotranslationally [25], different pathways were taken during synthesis to those taken when folding from a denatured state [54]. A further promising approach is found in [56]. Pathways which minimize the difficulty of folding to the native state (for example, those which avoid having the chain pass through an opening) are found; results indicate that earlier folding is more likely around the N-terminus than the C-terminus, so pointing to an asymmetry of the folding process that is confirmed in the current work.

Finally, there is also evidence of cotranslational protein folding that arises from numerical summaries of known protein structures. An analysis of structures in the Protein Data Bank (PDB) found that residues are, in general, closer to previously synthesized residues than those synthesized later, and that the N-terminal region was more compact than the C-terminal region [57]. It was argued that this provided evidence of cotranslational folding, however, these findings were contradicted by a later analysis of a larger set of proteins [58]. In the second study it was observed that the C-terminals were more compact and contained greater numbers of local contacts than N-terminals. Further analysis that considered topological accessibility (the ability of a protein to fold from a given residue as a starting point using only local contacts) found this to be more evident towards the N-terminus in the *α*/*β *class of proteins [59]. In a similar vein, Deane et al. [60] developed a measure of previous contacts which assesses the extent to which the chain forms contacts with previously extruded residues. They also found that the *α*/*β *class and ancient folds [61] exhibited such evidence of cotranslation.

To date, protein structure prediction methods do not incorporate cotranslational effects. This paper describes such an algorithm and evaluates its performance. This evaluation reveals that, in more than 94% of cases, a sequential algorithm that follows the sense of translation, that is, from N-terminus to C-terminus, is more accurate than an algorithm that follows the reverse sense, from C-terminus to N-terminus. The success of the sequential algorithm is greater the more the target shows evidence of cotranslational folding. It is also found that a sequential algorithm can match, and on occasion better (in 51% of proteins tested), the performance of a leading non-sequential protein structure prediction algorithm, namely Rosetta.

## Methods

### Structure prediction algorithms

A sequential algorithm (SAINT, a Sequential Algorithm Initiated at the Nitrogen Terminus) was developed and used to predict the structure of a number of proteins. This algorithm uses the Rosetta program [62] (version 2.1.0), extending it to incorporate cotranslational aspects of protein folding. To investigate the importance of following the direction of translation, the sequential algorithm was adapted to predict the structure of proteins produced in the reverse direction, from the C-terminus to the N-terminus. Predictions from the sequential and reverse sequential algorithms were compared and they in turn compared to predictions made using an unmodified version of Rosetta. These algorithms are now described.

#### Sequential algorithm

SAINT extends the peptide by a nine residue fragment at each iteration, starting with the N-terminus. Each fragment is added in a fully extended conformation (*ϕ *= -150°, *ψ *= 150° and *ω *= 180°). The final fragment may contain fewer than nine residues; it will contain as many residues as are required to complete the full protein chain. At each extension the peptide is allowed to fold and the conformation reached is used as the starting structure for the next extension, with Rosetta ab initio used to perform the structure predictions at each stage. In order to make comparisons between the sequential and non-sequential algorithms fair, each uses the same total number of cycles. For the sequential algorithm these cycles were distributed evenly amongst each extension of the peptide with the number of cycles calculated as follows. If *b *is a base number of cycles and *l *is the protein length then the total number of cycles *t *is *b*(*l*/100) and the number of extrusions *e *is ⌈*l*/9⌉. This results in *n *= ⌊*t*/*e*⌋ cycles for the first *e - *1 extrusions and *t *- *n*(*e *- 1) cycles for the final extrusion.

#### Reverse sequential algorithm

The reverse sequential algorithm is the same as the sequential algorithm. It differs only in that the peptide is extended from the C-terminus to the N-terminus.

#### Non-sequential algorithm

In non-sequential folding a protein is folded from a fully extended state. The Rosetta ab initio algorithm is employed for this process, using insertion from a library of fragments to build decoys (predicted structures). This has proved a successful technique for protein structure prediction in recent years [3,63-65]. Rosetta can select fragments from the target, so the algorithm as used here is not strictly ab initio. The number of cycles (fragment insertions) used by Rosetta varies with protein length in this study. A base number of 34,000 cycles was used for a protein of 100 residues, and this number increased proportionately; for example, for a protein with 143 residues the number of cycles is increased by a factor of 1.43. This is reasonable as in the cell longer proteins take more time to be synthesized, and thus have more time to explore conformational space before synthesis is completed.

### Selection of targets

In Deane et al. [60] a measure was developed, an Average Logarithmic Ratio (ALR), which assesses the extent of previous contacts within a peptide chain; proteins with positive ALR are expected to be those for which the cotranslational aspect of folding has a substantial impact, whilst proteins with negative ALR are expected to be those for which cotranslation has lesser impact. Two sets of targets were created from a PISCES[66] data set (*<*30% sequence identity, resolution better than 3 Å, at least 100 residues and no missing residues, downloaded 6 February, 2009). The first set contained protein chains with an ALR value of 0.15 or greater (total of 34 proteins), and the second contained chains with an ALR of -0.15 or less (total of 34 proteins); these two sets are referred to as the positive and negative sets respectively. For each protein in the two sets, 1000 decoys were generated with each of the algorithms described above (sequential, reverse sequential and non-sequential). GDT_TS values [67] were calculated for each of the resulting predictions. GDT_TS is defined as (*N*_1 _+ *N*_2 _+ *N*_4 _+ *N*_8_)/(4*N*), where *N*_*i *_is the number of corresponding residues within *i*Å and *N *is the total number of residues. It measures the closeness of corresponding residues in known and predicted structures, more heavily weighting closer pairs. It is helpful to see it in non-cumulative form as  where .

### Larger sample size

To establish whether the sample size (that is, the number of decoys produced for each protein) has an effect on the results, two proteins were subjected to a larger sampling. An additional 100,000 decoys were generated for the FLiG C-terminal domain of *Thermotoga maritima *(1qc7A) and also for 1ji4A, using the SAINT algorithm.

### Variability in peptide termini

As the differences between mean GDT_TS scores for SAINT and reverse SAINT, for a given protein, prove to be generally small, additional tests were conducted to ascertain whether terminus loop regions could be causing the observed effects. The termini of proteins are often unstructured, and their structure can be highly variable and difficult to predict. Small mistakes in the terminus regions could lead to the small differences observed between the mean GDT_TS scores.

The first N-terminus and last C-terminus secondary structure elements were identified in the experimental structure for each protein, and the termini up to the identified secondary structure element of the corresponding predicted model with the highest GDT_TS were removed. A secondary structure element was defined as a run of four residues with identical secondary structure assignment. Secondary structure was assigned from the experimentally determined structure with DSSP. In addition to these conditions the N-terminus and C-terminus secondary structure element had to be separated by at least five residues. GDT_TS scores were recalculated and counts taken of how often SAINT outperformed reverse SAINT and how often SAINT outperformed Rosetta.

### Clash analysis

A possible reason for better performance of SAINT was conjectured to be that extrusion from the nitrogen terminus produces fewer steric clashes than does extrusion from the carbon terminus. In order to investigate this, ten protein sequences were selected on the basis of their mean GDT_TS scores: four in which SAINT performed better, three in which reverse SAINT performed better, and three in which SAINT and reverse SAINT performed comparably. For each protein, two of the 1000 models generated were selected for each of SAINT and reverse SAINT. The extent of steric clashes in conformations following folding, for five extruded lengths (18, 36, 54, 72, 90), were assessed using MolProbity [68], a web server that calculates a "clashscore", equal to the number of steric overlaps that are greater than 0.4 Å per 1000 atoms. Nine residues in fully extended conformation were then added at the C-terminus (for SAINT) or the N-terminus (for reverse SAINT) to produce strings of length 27, 45, 63, 81, and 99 and these checked again for steric clashes. For each of the five positions, the clashscore before the addition of nine residues was subtracted from the clashscore after the addition of the 9-mer fragment. An average of the differences in clashscores, across all five lengths, was taken for each protein sequence and each algorithm.

### The importance of sense

To investigate why SAINT might perform consistently better than reverse SAINT, measures of secondary structure prediction quality were developed. For a given decoy, structural alignments for every overlapping fragment of 11 residues against the experimental structure were obtained, and the average C_*α*_-C_*α *_distance of the alignment was assigned to the fragment's center residue (fragments of 11 residues were chosen to provide insight into prediction accuracy on a more local scale than, for example, taking an entire secondary structure element). These residue-assigned distance measures were averaged across all residues in *α*-helices in the decoy (residue secondary structure was assigned by DSSP for the experimentally determined model) and these in turn averaged over all 1000 decoys. This was done for both the forward and reverse decoy sets. Finally, the forward helical prediction quality measure was subtracted from the reverse helical prediction quality measure. The same process was followed for *β*-strands. If directionality is not important in folding we would expect the accuracy of helical or strand predictions to be similar regardless of the direction of synthesis, resulting in the difference calculated above being zero. A positive difference would indicate that forward predictions were more accurate than reverse predictions while negative differences would indicate that reverse predictions were more accurate. One of the proteins in the positive set (1qc7A) and four in the negative set (1kf6D, 1mkaA, 1nekC and 1uz3A) contained no *β*-strand residues and, therefore, were not considered in the analysis.

## Results and Discussion

**Figure 1:**
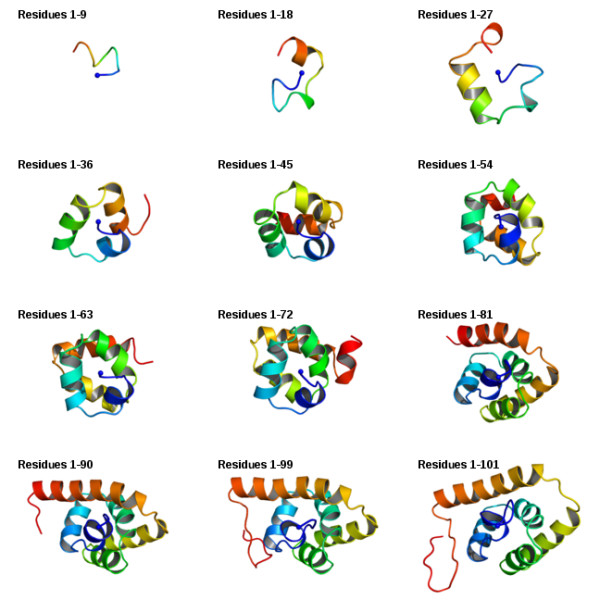
**Cotranslational structure prediction of the FLiG C-terminal domain **(1qc7A; **101 residues)**. Segments of nine residues are extruded at a time except for the last segment which consists of two residues. One thousand decoys were produced; the particular simulation above produced the structure with the highest GDT_TS of 63.12%. In each sub-figure the N-terminal is coloured dark blue and appears at the center adopting approximately the same orientation; it cannot always be the same orientation due to changes in conformation as the protein folds.

The emerging partial conformations produced by SAINT for sequence 1qc7A are shown in Figure 1, using the most successful decoy. The six helices are seen to progressively take shape as the chain is extruded, with early conformations largely preserved.

Results for SAINT, reverse SAINT and Rosetta for each of the proteins in the positive set (ALR ≥ 0.15, see Methods, Selection of targets) and negative set (ALR ≤ -0.15) are summarized in Table 1 and Table 2 respectively. The mean performance and best models produced by SAINT show that it predicts structures better than reverse SAINT in the majority of cases (Table 3). For example, SAINT yielded a higher mean GDT_TS than reverse SAINT for 32 of the 34 proteins with positive ALR and equally, for 32 of the 34 proteins with negative ALR.

**Table 1:** Results from positive set. Accuracy of models obtained for 34 proteins with ALR ≥ 0.15 using SAINT, reverse SAINT and Rosetta.

Code	Length	ALR	Mean GDT_TS			Maximum GDT_TS		
				
			SAINT	Reverse SAINT	Rosetta	SAINT	Reverse SAINT	Rosetta
1bmtA	246	0.1509	17.39	*14.44*	**17.50**	**30.28**	*24.19*	26.12
1hjrA	158	0.1777	21.56	*19.06*	**21.75**	**41.77**	*30.06*	35.76
1ji4A	144	0.1851	*30.37*	32.61	**32.77**	49.31	*48.09*	**50.17**
1k5nA	276	0.1997	10.96	*10.58*	**11.03**	16.94	**17.21**	*15.58*
1mf7A	194	0.2106	**18.17**	*15.08*	18.15	28.74	*27.06*	**31.31**
1n2zA	245	0.1668	14.04	*12.05*	**14.12**	20.41	*17.24*	**21.43**
1oaaA	259	0.1909	**20.41**	*14.51*	19.11	**35.14**	*25.97*	32.14
1qc7A	101	0.2762	39.69	*34.93*	**41.31**	**63.12**	61.63	*55.94*
1ryp2	233	0.2030	14.74	*13.83*	**15.13**	22.75	*20.71*	**26.07**
1rypI	222	0.3251	**15.37**	*13.69*	15.21	24.21	*21.28*	**24.77**
1tcaA	317	0.1592	**11.32**	*8.58*	10.70	19.32	*15.69*	**19.56**
1wehA	171	0.1635	19.21	*18.56*	**19.36**	**32.89**	*28.22*	31.14
1y1lA	124	0.2226	22.34	*21.63*	**23.20**	**36.69**	*33.27*	36.49
1yqgA	263	0.1723	**17.23**	*13.66*	17.04	26.62	*21.77*	**27.09**
1yw5A	177	0.1637	17.36	*16.41*	**17.96**	26.69	*24.15*	**27.26**
1zxxA	319	0.1576	**11.67**	*9.73*	11.63	**19.20**	*15.75*	17.87
2d00A	109	0.2345	**31.79**	*23.93*	31.22	**49.77**	*42.20*	47.25
2d1pB	119	0.1581	23.65	*21.26*	**24.29**	**38.03**	*32.56*	36.13
2ehgA	149	0.2088	21.74	*19.51*	**21.80**	**44.97**	*30.54*	32.72
2euiA	153	0.2054	22.07	*21.29*	**22.67**	38.73	*36.76*	**40.20**
2f1kA	279	0.1664	**16.75**	*14.49*	16.39	**28.23**	*21.68*	27.78
2g64A	140	0.1676	19.86	*18.55*	**20.66**	29.64	*27.50*	**30.54**
2h0rA	216	0.1555	*13.77*	**15.35**	14.57	*21.18*	23.03	**27.78**
2hy5A	130	0.1693	23.39	*21.54*	**23.60**	**37.12**	*30.38*	36.73
2imfA	203	0.1810	18.34	*16.25*	**18.41**	28.20	**28.33**	*25.00*
2j01V	101	0.1604	**20.27**	*18.26*	20.12	27.97	*26.49*	27.97
2jdjA	105	0.1666	23.39	*21.53*	**24.07**	39.05	*35.00*	**45.71**
2ocgA	254	0.1793	16.33	*11.62*	**16.45**	**24.31**	*21.75*	23.92
2pd2A	108	0.2397	30.66	*28.83*	**30.82**	51.62	*49.54*	**54.86**
2q35A	243	0.2346	13.77	*13.24*	**13.98**	**23.05**	*19.14*	20.37
2rcyA	262	0.1922	**16.71**	*14.10*	16.67	**26.15**	*21.18*	24.62
2rhwA	283	0.1538	12.66	*10.58*	**13.52**	**21.73**	*17.67*	21.20
3beoA	375	0.1637	10.18	*8.42*	**10.21**	15.93	*13.67*	**16.07**
3vubA	101	0.1550	**25.75**	*22.37*	25.62	**67.57**	*37.62*	51.24

The mean GDT_TS and maximum GDT_TS for all 1000 decoys produced for each combination of protein and algorithm is shown. For both the mean and maximum GDT_TS the highest GDT_TS is shown in bold while the lowest is shown in italics.

**Table 2:** Results from negative set. Accuracy of models obtained for 34 proteins with ALR ≤ -0.15 using SAINT, reverse SAINT and Rosetta.

Code	Length	ALR	Mean GDT_TS			Maximum GDT_TS		
				
			SAINT	Reverse SAINT	Rosetta	SAINT	Reverse SAINT	Rosetta
1aocA	175	-0.2193	14.48	*14.41*	**14.96**	**21.57**	*19.57*	20.43
1aym1	285	-0.2877	**7.46**	*7.07*	7.40	10.26	*9.56*	**10.79**
1aym3	238	-0.1526	9.19	*7.92*	**9.26**	13.97	*10.71*	13.97
1ddlA	188	-0.2148	10.87	*10.50*	**10.95**	16.09	*15.69*	**17.69**
1dwkA	156	-0.1839	20.23	*18.97*	**20.29**	*32.05*	32.37	**33.17**
1dy5A	124	-0.1685	17.07	*16.77*	**17.48**	**26.41**	25.60	*25.40*
1e0cA	271	-0.1927	11.53	*9.48*	**12.36**	16.61	*13.01*	**18.82**
1kf6D	119	-0.1764	25.06	*24.13*	**25.44**	38.03	**38.66**	*34.45*
1kptA	105	-0.1756	22.50	*21.20*	**22.91**	**31.67**	*28.57*	30.71
1kyfA	247	-0.2037	12.67	*9.60*	**13.26**	20.34	*18.93*	**20.65**
1l7lA	121	-0.1779	15.17	*13.81*	**15.90**	20.87	*20.25*	**22.11**
1mkaA	171	-0.1794	*15.88*	16.32	**16.48**	*23.98*	25.15	25.15
1nekC	129	-0.2053	27.71	*26.88*	**28.98**	44.77	*42.05*	**45.93**
1p0zA	131	-0.1594	31.27	*27.99*	**33.13**	42.75	*40.84*	**58.21**
1qqp3	220	-0.3876	10.10	*8.60*	**10.13**	**16.25**	*11.70*	14.77
1seiA	130	-0.2636	**25.49**	*20.64*	24.06	40.77	*35.77*	40.77
1tt8A	164	-0.1881	16.36	*13.53*	**17.02**	24.54	*23.63*	**25.46**
1umhA	184	-0.1630	11.68	*10.31*	**11.83**	**17.93**	16.71	*16.58*
1uz3A	102	-0.1711	*28.90*	**31.22**	29.49	41.42	**43.87**	*39.46*
1wt9B	123	-0.1723	**21.70**	*18.90*	21.60	**37.20**	*29.88*	30.49
1y8cA	246	-0.1984	**15.77**	*11.56*	15.09	**27.54**	*19.51*	23.98
2ag4A	164	-0.2084	13.61	*11.99*	**13.86**	*19.66*	19.82	**20.58**
2awgA	118	-0.1693	19.78	*16.46*	**20.02**	29.45	*26.48*	**32.42**
2b0aA	186	-0.1747	13.33	*11.94*	**13.62**	**20.97**	*18.15*	19.49
2bnqD	203	-0.1799	**13.29**	*9.90*	13.16	**25.12**	*18.35*	20.94
2e56A	144	-0.1542	14.06	*13.85*	**14.21**	**21.53**	19.27	19.27
2edmA	161	-0.1638	11.45	*11.33*	**11.77**	16.61	**16.77**	*16.46*
2nwfA	141	-0.1601	20.39	*17.20*	**21.93**	**34.04**	*29.79*	33.51
2ov0A	105	-0.2059	19.70	*17.86*	**20.37**	*27.62*	**30.24**	30.00
2owpA	129	-0.1827	22.61	*21.33*	**23.30**	34.69	**35.47**	34.69
2p25A	126	-0.1604	28.74	*27.95*	**30.79**	46.03	*43.45*	**55.75**
2tgiA	112	-0.2279	18.38	*17.64*	**18.79**	*24.55*	26.56	**27.46**
3besR	250	-0.1606	11.54	*10.88*	**11.85**	**17.80**	*16.80*	17.40
3ezmA	101	-0.3241	28.74	*20.25*	**29.04**	**38.12**	36.14	36.14

The mean GDT_TS and maximum GDT_TS for all 1000 decoys produced for each combination of protein and algorithm is shown. For both the mean and maximum GDT_TS the highest GDT_TS is shown in bold while the lowest is shown in italics.

**Table 3:** Summary of results. Pairwise (SAINT vs reverse SAINT and SAINT vs Rosetta) comparison of the algorithms.

	Mean		Maximum	
	
	Positive	Negative	Positive	Negative
SAINT	32 ***	32 * * *	32 * * *	25 **
Reverse SAINT	2	2	2	9
				
SAINT	11	5	19	16
Rosetta	23 *	29 * * *	15	14

The table shows the number of times an algorithm in a pair outperformed the other, separately for the positive and negative sets. Both mean GDT_TS and maximum GDT_TS are used as measures of performance. Asterisks indicate binomial test *p*-values where * is < 0.05, ** is < 0.01, *** is < 0.001.

**Figure 2:**
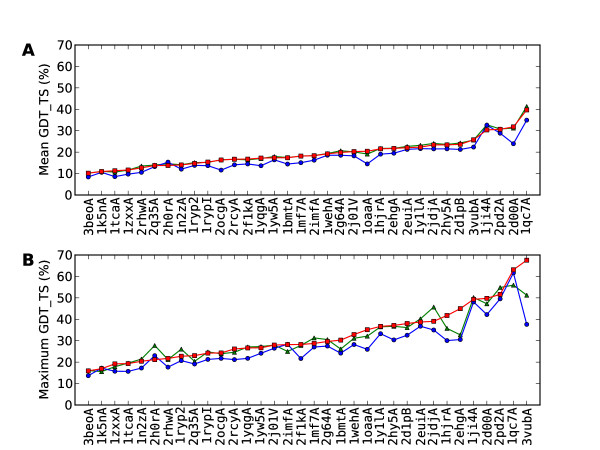
**Plots of mean and maximum GDT_TS for the positive set**. Graphic **A **shows the mean GDT_TS scores for the 34 proteins in the positive set, for SAINT (red squares), reverse SAINT (blue circles) and Rosetta (green triangles), with the proteins ordered according to ascending mean SAINT GDT_TS. SAINT and Rosetta perform similarly and consistently better than reverse SAINT. Graphic **B **plots maximum GDT_TS in the same way, ordered this time by ascending maximum SAINT GDT_TS, revealing greater variation but still a consistent and generally larger improvement of SAINT on reverse SAINT.

**Figure 3:**
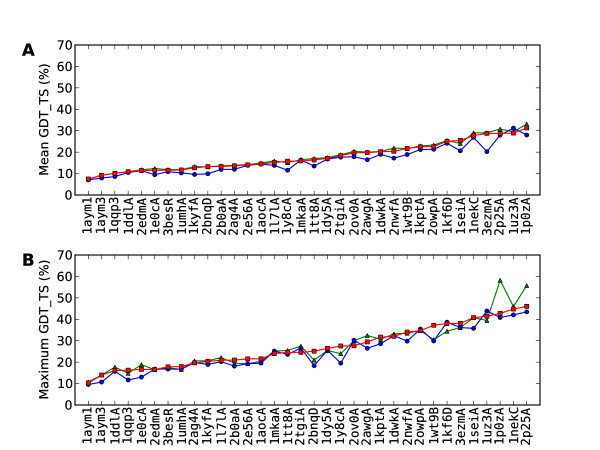
**Plots of mean and maximum GDT_TS for the negative set**. Graphic **A **shows the mean GDT_TS scores for the 34 proteins in the negative set, for SAINT (red squares), reverse SAINT (blue circles) and Rosetta (green triangles), with the proteins ordered according to ascending mean SAINT GDT_TS. Graphic **B **plots maximum GDT_TS for proteins in the negative set, ordered by ascending maximum SAINT GDT_TS. Outcomes are the same as for the positive set, with all differences less marked.

Plots of the mean scores for SAINT, reverse SAINT and Rosetta for the positive set are given in Figure 2A, with proteins ordered from smallest to largest mean SAINT GDT_TS score. Corresponding plots for the negative set are given in Figure 3A. The consistent superiority of SAINT over reverse SAINT is evident, with the difference being slightly greater for the positive set. The largest such difference seen in all the data is 8.49%, observed between the means of SAINT and reverse SAINT for 3ezmA (negative set), and representing an increase in GDT_TS from 20.25% to 28.74%. Mean performances of SAINT and Rosetta indicate that Rosetta outperforms SAINT in both the positive (Rosetta 19.72, SAINT 19.50) and negative (Rosetta 18.26, SAINT 17.84) sets. The difference is greater for the negative set (Table 3).

**Figure 4:**
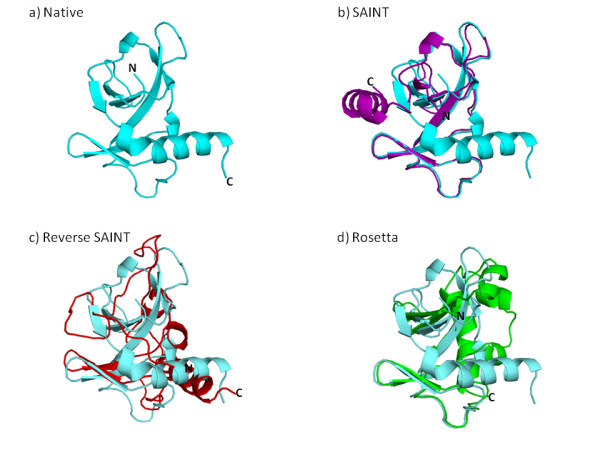
**Superpositions of the best predictions for **3vubA** on the native structure**. The best decoy produced overall was by SAINT for 3vubA, whose native conformation is shown in a). The remaining graphics show the superposition of this native conformation with the best decoy produced by b) SAINT (GDT_TS = 67.57), c) reverse SAINT (GDT_TS =37.62) and d) Rosetta (GDT_TS = 51.24). The SAINT decoy best captures the native loop and sheet conformation; a loop error causes the C-terminal helix to be incorrectly oriented.

Plots of the maximum scores for SAINT, reverse SAINT and Rosetta for the positive set are given in Figure 2B, with proteins ordered from smallest to largest maximum SAINT GDT_TS score. Corresponding plots for the negative set are shown in Figure 3B. When considering best performance, SAINT is again superior to reverse SAINT, and more so in the positive set. Rosetta is no longer superior when best performance is considered; SAINT outperforms Rosetta, for example, in 19 of the 34 proteins in the positive set. The most successful SAINT prediction in the positive set was found for 3vubA. It is shown superposed on the native conformation in Figure 4, together with superpositions of the best reverse SAINT and Rosetta predictions on the native conformation. SAINT captures the structure better than either reverse SAINT or Rosetta.

A GDT_TS value of 30% or above is generally considered to ensure that a reasonable prediction is found [4]; a scan of Table 1 indicates that roughly one half (15 out of 34) of the best SAINT predictions are satisfactory, and similarly for Rosetta (16 out of 34).

### Larger sample size

Summaries of the distribution of GDT_TS scores indicate that the size of the decoy sets used (that is, 1000) does not significantly influence their values (for 1qc7A, sample size of 1000 has min. 23.0, max. 69.8, mean 40.6, std devn 7.9; sample size of 100,000 has min. 22.0, max. 73.0, mean 40.9, std devn 8.2). When repeated with 1ji4A, similar results were produced (sample size of 1000 has min. 19.79, max. 49.31, mean 30.37, std devn 4.07; sample size of 100,000 has min. 17.71, max. 56.94, mean 30.78, std devn 4.38).

### Variability in peptide termini

The results of this test indicate that the differences in GDT_TS observed are not due to variability in the terminus regions of the peptides (data presented in Tables 4 and 5).

**Table 4:** Variability in peptide termini: Results from positive set.

Code	Length	ALR	Maximum GDT_TS		
			
			SAINT	Reverse SAINT	Rosetta
1bmtA	246	0.1509	**31.28**	*24.79*	27.23
1hjrA	158	0.1777	**42.33**	*31.00*	37.67
1ji4A	144	0.1851	50.18	*48.75*	**50.71**
1k5nA	276	0.1997	18.08	**18.37**	*16.35*
1mf7A	194	0.2106	29.05	*27.97*	**31.76**
1n2zA	245	0.1668	20.26	*18.21*	**20.37**
1oaaA	259	0.1909	**35.60**	*25.70*	32.90
1qc7A	101	0.2762	**70.12**	67.38	*62.20*
1ryp2	233	0.2030	23.98	*21.25*	**27.39**
1rypI	222	0.3251	27.65	*25.13*	**29.50**
1tcaA	317	0.1592	22.27	*18.09*	**22.45**
1wehA	171	0.1635	**33.18**	*28.87*	31.55
1y1lA	124	0.2226	**36.67**	*32.71*	35.83
1yqgA	263	0.1723	26.74	*22.10*	**27.41**
1yw5A	177	0.1637	28.81	*25.91*	**29.42**
1zxxA	319	0.1576	**19.60**	*15.95*	18.10
2d00A	109	0.2345	**53.32**	*45.92*	50.77
2d1pB	119	0.1581	**39.13**	*31.96*	36.96
2ehgA	149	0.2088	**46.03**	*31.03*	33.62
2euiA	153	0.2054	42.88	*41.54*	**45.96**
2f1kA	279	0.1664	**28.75**	*22.34*	28.02
2g64A	140	0.1676	31.44	*29.17*	**32.58**
2h0rA	216	0.1555	*20.05*	20.89	**25.85**
2hy5A	130	0.1693	**37.60**	*31.10*	37.20
2imfA	203	0.1810	28.55	**29.19**	*25.63*
2jdjA	105	0.1666	41.84	*38.16*	**50.26**
2ocgA	254	0.1793	**24.20**	*21.71*	**24.20**
2pd2A	108	0.2397	52.12	*50.47*	**55.42**
2q35A	243	0.2346	**23.52**	*19.09*	20.46
2rcyA	262	0.1922	**25.79**	*21.75*	24.51
2rhwA	283	0.1538	**21.88**	*17.77*	21.25
3beoA	375	0.1637	16.41	*14.29*	**16.48**
3vubA	101	0.1550	**70.31**	*39.06*	52.34

Among the 1000 decoys produced for each protein with ALR ≥ 0.15 by each of SAINT, reverse SAINT, and Rosetta the best model (with highest GDT_TS) was found (as indicated in Table 1 by Maximum GDT_TS). Each of these selected models was then altered by chopping off the first N-terminus and last C-terminus secondary structure elements identified in its native structure. GDT_TS scores were then recalculated for each algorithm and are displayed below. The highest GDT_TS is shown in bold while the lowest is shown in italics. Sample size was reduced to 33 as no secondary structural element at least five residues in length was found at either terminal of the protein chain 2j01Vpdb2j01V.

**Table 5:** Variability in peptide termini: Results from negative set.

Code	Length	ALR	Maximum GDT_TS		
			
			SAINT	Reverse SAINT	Rosetta
1aocA	175	-0.2193	**24.83**	*20.72*	23.12
1aym1	285	-0.2877	11.54	*10.02*	**12.45**
1aym3	238	-0.1526	15.32	*11.75*	**15.55**
1ddlA	188	-0.2148	19.24	19.24	**22.04**
1dwkA	156	-0.1839	34.48	*33.62*	**35.52**
1dy5A	124	-0.1685	**26.46**	*25.00*	26.25
1e0cA	271	-0.1927	16.97	*14.26*	**20.38**
1kf6D	119	-0.1764	41.51	**42.45**	*38.21*
1kptA	105	-0.1756	**34.84**	*30.32*	32.98
1kyfA	247	-0.2037	20.27	*18.80*	**20.48**
1l7lA	121	-0.1779	21.37	*20.94*	**22.65**
1mkaA	171	-0.1794	*25.64*	**26.91**	25.96
1nekC	129	-0.2053	**54.21**	*49.07*	53.97
1p0zA	131	-0.1594	46.01	*43.49*	**60.92**
1qqp3	220	-0.3876	21.20	*15.06*	18.86
1seiA	130	-0.2636	**41.47**	*36.31*	41.27
1tt8A	164	-0.1881	25.32	*24.52*	**26.61**
1umhA	184	-0.1630	**18.37**	16.99	*16.85*
1uz3A	102	-0.1711	49.41	**51.47**	*45.00*
1wt9B	123	-0.1723	**35.81**	30.18	*27.25*
1y8cA	246	-0.1984	**27.67**	*19.52*	24.38
2ag4A	164	-0.2084	*20.09*	20.41	**21.04**
2awgA	118	-0.1693	31.65	*25.46*	**33.26**
2b0aA	186	-0.1747	**23.33**	*20.15*	21.97
2bnqD	203	-0.1799	**26.24**	*20.44*	22.93
2e56A	144	-0.1542	**22.01**	*19.40*	20.34
2edmA	161	-0.1638	**18.28**	**18.28**	*18.10*
2nwfA	141	-0.1601	**35.04**	*29.56*	34.31
2ov0A	105	-0.2059	*29.17*	**31.25**	30.95
2owpA	129	-0.1827	36.67	**37.29**	*35.42*
2p25A	126	-0.1604	48.08	*46.37*	**58.12**
2tgiA	112	-0.2279	*26.21*	28.88	**29.61**
3besR	250	-0.1606	**17.58**	*16.36*	16.77
3ezmA	101	-0.3241	**37.37**	36.86	*35.82*

Among the 1000 decoys produced for each protein with ALR ≤ -0.15 by each of SAINT, reverse SAINT, and Rosetta the best model (with highest GDT_TS) was found (as indicated in Table 2 by Maximum GDT_TS). Each of these selected models was then altered by chopping off the first N-terminus and last C-terminus secondary structure elements identified in its native structure. GDT_TS scores were then recalculated for each algorithm and are displayed below. The highest GDT_TS is shown in bold while the lowest is shown in italics.

### Clash analysis

The results are shown in Table 6. Four of the ten protein conformations examined have higher steric clashscores for SAINT than reverse SAINT. The steric clashscore appears not to be influenced by its mean GDT_TS score, evidenced by two (1mf7A and 2d00A) out of the four proteins with higher mean GDT_TS scores for SAINT having greater steric clashscores than reverse SAINT. Steric clashes produced by SAINT and reverse SAINT are generally comparable, so providing no evidence that fewer steric clashes are the reason for the better performance of SAINT.

**Table 6:** Clash analysis.

	Code	Forward SAINT mean	Reverse SAINT mean
SAINT better	1mf7A	18.894	1.525
	1oaaA	-2.441	4.579
	2d00A	13.922	-4.265
	1qc7A	-5.440	2.238

Reverse SAINT better	1ji4A	-8.578	-5.016
	1uz3A	-7.861	29.370
	2h0rA	3.683	-6.645

SAINT and Reverse SAINT comparable	1aocA	-1.327	-3.650
	1kf6D	-8.691	-1.861
	2edmA	-6.029	-0.610

Mean difference in clashscores for each protein sequence; the larger the mean difference, the more clashes created by the extrusion. The first four proteins in the table have higher mean GDT_TS scores for SAINT, the next three have higher mean GDT_TS scores for reverse SAINT and the remaining three have comparable mean GDT_TS scores for SAINT and reverse SAINT. There is no evidence that SAINT creates more clashes.

### The importance of sense

**Figure 5:**
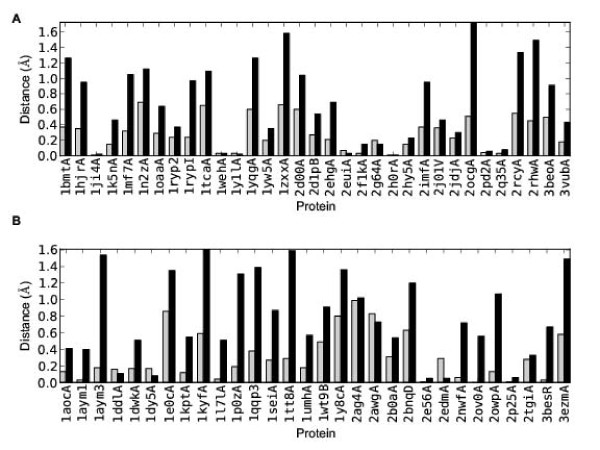
**Accuracy of helix and strand predictions**. Accuracy of helix and strand predictions separately for (A) positive and (B) negative sets. Plots show the difference (reverse SAINT minus SAINT) in the secondary structure distance measure for helical (grey) and strand (black) residues. Positive values here indicate that SAINT is producing predictions that are more accurate than those of reverse SAINT. Evidently SAINT outperforms reverse SAINT for both types of secondary structure, but more strongly for strands and the negative set.

The differences obtained from both the positive and negative sets are shown in Figure 5. These results show that for both types of secondary structure SAINT is generally producing better predictions, but that the difference is more pronounced for strand residues. In 28 of the 33 proteins (85%) in the positive set the difference between forward and reverse folding is greater for strands than for helices (with 16 (48%) having a *β*-strand difference more than twice the *α*-helix difference). Similarly, in 26 of the 30 proteins (87%) in the negative set the difference between forward and reverse folding is greater for strands than for helices (with 19 (63%) having a *β*-strand difference more than twice the *α*-helix difference). These results indicate that in general SAINT is more accurate when predicting strands than is reverse SAINT. These differences are small, but they would account for the differences observed in the results.

## Discussion

A consistent difference in prediction accuracy was seen between SAINT and reverse SAINT. SAINT is markedly superior to reverse SAINT, and slightly more so for proteins with positive ALR values. When looking in detail at SAINT and reverse SAINT, the differences observed are most likely due to the detrimental effect on strand prediction observed when elongating a peptide from the C-terminus to the N-terminus. SAINT produced decoys with a higher mean GDT_TS than reverse SAINT for more than 94% of proteins in both the positive and negative protein sets. The differences between mean GDT_TS scores for SAINT and reverse SAINT decoys were also bigger than those between SAINT and Rosetta decoys. If directionality played no part in the folding process it would be expected that there would be no difference in the predictive accuracy of extrusions from the N-terminus to C-terminus and extrusion from C-terminus to the N-terminus. Three possible explanations for these results are outlined below.

Peptides, when extruded from the ribosome, start at the N-terminus. For this reason, fragments near the N-terminus are less influenced in their folding by the remainder of the peptide, since this has yet to emerge from the ribosome. On the other hand, fragments towards the C-terminus must fold in the presence of the bulk of the peptide. Thus the conformation assumed by the early fragment is a local choice, in that it depends largely on the amino acid sequence of the fragment. The conformation reached by a later fragment is determined by more than its amino acid sequence, in that it also depends on surrounding structure. This behaviour is mimicked by SAINT but not by reverse SAINT, so providing an explanation for the consistently better predictive accuracy of SAINT.

A second explanation arises from the way that the two algorithms allocate fragment insertions. At any stage, due to the constraints of Rosetta, fragment insertions are made uniformly across the currently extruded peptide length. The upshot is that more fragment insertions are attempted at the N-terminus than the C-terminus for SAINT while the opposite is true for reverse SAINT. Should it be the case that the N-terminus of the peptide is harder to predict than the C-terminus, SAINT would be more successful than reverse SAINT since SAINT puts in effort where it is needed. Due to the reasons stated above, however, we expect the N-terminus to be more easily predicted than the C-terminus.

A third possibility is that Rosetta itself has some inherent directionality, so favouring SAINT over reverse SAINT. A study of Rosetta, however, provides no indication of such a directional bias.

A strong correlation between mean GDT_TS and chain length is seen for both the positive and negative sets and for all three algorithms: as the chain length increases the GDT_TS decreases. 1oaaA is the only target over 200 residues in length that produced a set of decoys with mean GDT_TS greater than 20%, indicating that the versions of the algorithms employed in this study are not sufficient to accurately predict the structure of chains with more than 200 residues (this accounts for 50% of the positive set and 24% of the negative set). Excluding this data from the analysis, however, makes no difference to the overall findings.

Given that SAINT outperforms reverse SAINT it might be expected that SAINT would also outperform Rosetta, Rosetta being, in some senses, midway between the two. In best performance, arguably more important than mean performance, there is weak evidence that SAINT does outperfom Rosetta; for the positive set SAINT outperfoms Rosetta in 19 out of 33 instances (there is one tie) and for the negative set SAINT outperforms Rosetta in 16 out of 30 instances (there are four ties). An explanation why this remains weak at this stage is that SAINT remains crude, barely exploiting spatial and temporal advantages which may be available in cotranslational folding; we have simply used an iterative version of Rosetta. For example, at each extrusion, fragment insertions are chosen uniformly along the extruded peptide, whereas use of an insertion location distribution skewed towards the carbon terminus might be more realistic. To its credit, however, the SAINT versus reverse SAINT investigation exploits the power of a "paired comparison" design more effectively than does the SAINT versus Rosetta investigation, in that it contrasts opposites and so is more likely to reveal an effect.

## Conclusions

This study has presented an algorithm that builds cotranslation into protein structure prediction. To assess the importance of the direction of translation the sequential algorithm was compared to a reverse sequential algorithm where the protein was produced from the C-terminus to N-terminus. Two sets of proteins were chosen: one where the residues have, on average, more contacts with previous residues than successive residues and the other where the residues have, on average, more contacts with successive residues than previous residues. The performance of the sequential algorithm for protein structure prediction was also compared with Rosetta, which folds from a fully elongated chain.

When SAINT was compared to reverse SAINT a very pronounced difference was observed. When mean GDT_TS was used as the performance measure SAINT outperformed reverse SAINT for over 94% of targets from both the positive and negative sets. These figures were still high when the maximum GDT_TS was used as the performance measure, with SAINT outperforming reverse SAINT in over 91% of targets from the positive set and over 73% of targets from the negative set.

The results show that Rosetta produces decoy sets with higher mean GDT_TS scores than SAINT for both the positive and negative protein sets, but that this superiority of Rosetta is not seen when the models with the highest GDT_TS scores are compared. If it were possible to always select the most accurate structure from the set of decoys then SAINT would, overall, produce a better prediction than Rosetta. The selection of the best decoy from a set, however, is a separate problem that is not addressed in this study. While Rosetta is producing decoy sets with higher mean GDT_TS scores than SAINT, examination of the differences between the means shows that the difference is always small. Only on one occasion does a Rosetta decoy set have a mean GDT_TS greater than 2% above the corresponding SAINT decoy set (an increase in mean GDT_TS from SAINT to Rosetta of 2.4% for 1ji4A). It has been established that the size of the decoy set and flexibility of peptide terminus residues do not affect the distribution of GDT_TS scores.

The sequential algorithm described in this study is in its earliest stages of development. Future work will include investigation of the effect of translation speed, allowing extruded segments to have variable length and the number of fragment insertion attempts at each iteration to vary. Improvements should also include incorporation of spatial restrictions to simulate the constraint of the ribosome tunnel.

## Authors' contributions

Conceived and designed the experiments: FPEH, GRW, CMD and JJE. Performed the experiments: JJE, FPEH and SS. Analyzed the data: JJE, GRW, FPEH and CMD and SS. Wrote the paper: JJE, FPEH, GRW, SS and CMD. All authors read and approved the final manuscript.
